# Computed Tomography Measurement of Hepatic Steatosis: Prevalence of Hepatic Steatosis in a Canadian Population

**DOI:** 10.1155/2016/4930987

**Published:** 2016-04-19

**Authors:** Malcolm M. Wells, Zhe Li, Bryan Addeman, Charles A. McKenzie, Amol Mujoomdar, Melanie Beaton, Jeffery Bird

**Affiliations:** ^1^Department of Medicine, Division of Digestive Care and Endoscopy, Dalhousie University, Victoria General Hospital, 1276 South Park Street, Halifax, NS, Canada B3H 2Y9; ^2^Schulich School of Medicine and Dentistry, Western University, 1465 Richmond Street, London, ON, Canada N6G 2M1; ^3^Department of Medical Biophysics, Schulich School of Medicine and Dentistry, Western University, 1151 Richmond Street N., London, Ontario, Canada N6A 5C1; ^4^Department of Medical Imaging, Schulich School of Medicine and Dentistry, Western University, London Health Sciences Centre, Victoria Hospital, London, ON, Canada N6A 5W9; ^5^Department of Medicine, Division of Gastroenterology and Hepatology, Schulich School of Medicine and Dentistry, Western University, London, ON, Canada N6A 5A5; ^6^Central Alberta Medical Imaging Services Ltd., 4312 54 Avenue, Red Deer, AB, Canada T4N 4M1; ^7^Department of Medical Imaging, Red Deer Regional Hospital, 3942 50a Avenue, Red Deer, AB, Canada T4N 4E7

## Abstract

*Background/Aims*. Nonalcoholic fatty liver disease (NAFLD) is a chronic liver disease that can progress to cirrhosis and hepatocellular carcinoma. This retrospective chart review investigated the incidence of hepatic steatosis in London, Ontario, Canada.* Methods*. A retrospective chart review was performed on emergency room (ER) patients undergoing nonscheduled computed tomography (CT) imaging over a six-month period in London, Ontario. CT images and reports were examined to determine presence of steatosis. Analyses of the electronic chart for a period of six months following the CT and communication with the patients' family doctors were used to determine if there was follow-up. Waist circumference, subcutaneous fat depth, and abdominal fat volumes were calculated.* Results*. 48/450 patients meeting inclusion criteria were identified by radiology as having steatosis, with 34/40 (85%) family physicians unaware of the finding. 24.7% (100/405) of patients met standard CT criteria for steatosis, 40 of which were reported by the radiologist. Waist circumference, subcutaneous adipose tissue depth, subcutaneous adipose tissue volume, and visceral adipose tissue volume were significantly associated with steatosis.* Conclusions*. The hepatic steatosis prevalence we report is the first reported in a Canadian population. Early identification of steatosis will become more important as new pharmacologic therapies arise.

## 1. Introduction

With the rapidly growing prevalence of obesity throughout the world [1], morbidity and mortality related to its complications are on the rise [2]. As obesity rates continue to rise in North America, nonalcoholic fatty liver disease (NAFLD) has become an epidemic. NAFLD has a spectrum of disease severity, from simple steatosis to steatohepatitis, with potential progression to fibrosis and cirrhosis [3, 4]. A small portion of patients with cirrhosis will develop hepatocellular carcinoma [5]. The frequency of liver transplantation for patients with NAFLD-related cirrhosis has increased fivefold over the last ten years [6]. NAFLD-related cirrhosis is currently the second most common indication for liver transplantation and is projected to become the most common indication for liver transplantation by 2020 [6].

Establishing a definitive diagnosis of NAFLD requires both clinical and histologic data. However, a minority of patients receive parenchymal liver biopsies. Instead, the combination of patient history, physical examination, blood test results, and radiologic findings is used to exclude other causes of liver disease. Imaging studies support the diagnosis. CT diagnostic criteria for steatosis are liver attenuation at least 10 Hounsfield Units (HU) less than that of the spleen or absolute liver attenuation of less than 40 HU. Unenhanced CT has a sensitivity for steatosis ranging from 43 to 95% and a specificity of 90–100% [7, 8]. Sensitivity rises to 93% for detecting steatosis involving greater than 33% of the liver, with positive predictive value of 76% [9]. With contrast-enhanced CT, a difference of 18.5 HU between liver and spleen attenuation had a sensitivity of 93%, a specificity of 93%, and a receiving operating curve of 0.98 [10]. The sensitivity and specificity of unenhanced CT are similar to those of ultrasound (SN 84.8%, SP 93.6%) and Magnetic Resonance Imaging (SN 81%, SP 100%) [7, 8].

Visceral abdominal tissue (VAT) is associated with cardiovascular disease [11–13] and its risk factors, including diabetes mellitus [14–16], insulin resistance [15, 17, 18], hypertension [19–21], dyslipidemia [22–24], and metabolic syndrome [11–13, 25]. NAFLD is closely tied to metabolic syndrome, diabetes mellitus type 2, and cardiovascular disease [26, 27]. Visceral fat, which releases portal free fatty acids and secretes adipokines, particularly adiponectin, is central to NAFLD's pathophysiology [28].

The aim of the current study was to determine the prevalence of hepatic steatosis, determine the follow-up of the incidental finding of hepatic steatosis, and characterize the accuracy of radiologic reporting of hepatic steatosis. Furthermore, we aimed to determine an association between steatosis and quantitative measures of waist circumference and abdominal adipose tissue.

## 2. Methods

### 2.1. Study Subjects

A retrospective chart review was performed on all computed tomography (CT) Scans of the thorax, abdomen, and pelvis performed at the London Health Science Centre (LHSC) during “on-call” hours (between the hours of 5:00 pm and 8:00 am, Monday to Friday, and between 8 am and 8 am, Saturday and Sunday) between January 1, 2011, and July 31, 2011. Patients were excluded if the CT was scheduled in advance, if the indication was for elevated liver enzymes or known liver pathology, and if the patient was admitted to hospital following the CT Scan. The CT studies obtained during “on-call” hours were used as there was a readily available database with focused clinical history and exam findings. Patients had to be undergoing imaging for a primary complaint felt to be independent of liver pathology, as to provide a representative sample of the general population. Patients were required to be local residents of London, Ontario, Canada, to ensure documentation of follow-up was complete.

### 2.2. Determination of the Incidental Findings of Fatty Liver Disease and Patient Liver Function

The official, signed electronic radiology reports were screened for incidental findings of fatty liver disease. Patients who were reported to have fatty liver disease on their ER CT had an electronic chart review performed, documenting ER bloodwork, including alanine aminotransferase (ALT), aspartate aminotransferase (AST), alkaline phosphatase (ALP), gamma glutamyl transferase (GGT), bilirubin, platelet count, and International Normalized Ratio (INR).

### 2.3. Follow-Up Assessment of Patients with Incidental Findings of Fatty Liver Disease

The electronic charts of patients having incidental findings of fatty liver disease were reviewed to assess patient follow-up of fatty liver disease. Patient follow-up was considered to have occurred if there was any consult made to either the family physician or a specialist to further assess and manage the patient's possible fatty liver disease.

The family physicians of patients who were reported to have the incidental finding of fatty liver disease were contacted via phone to determine if follow-up had been arranged. Phone attempts were made until follow-up was determined.

### 2.4. Measurement of Liver and Spleen Attenuation

**Figure 1:**
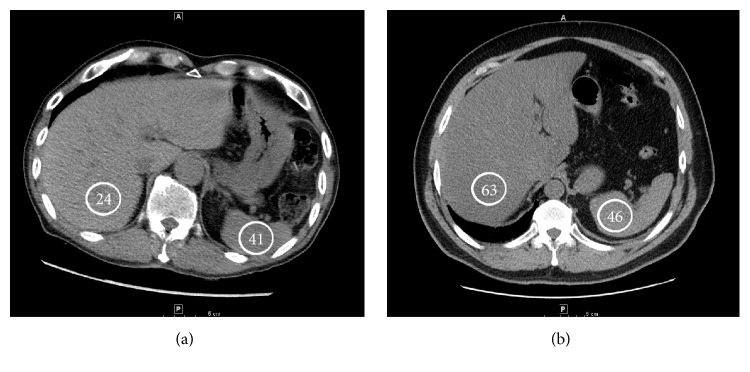
CT images showing an example of steatosis (a) and a nonsteatotic liver (b). Hounsfield Units are indicated in the circles.

CT criteria for steatosis on non-intravenous contrast scans included the following: liver attenuation 10 HU less than the spleen attenuation, absolute liver attenuation of less than 40 HU, and liver-to-spleen attenuation ratio less than 1 [7, 8]. An example of liver and spleen attenuation measurement is shown in Figure 1. CT Pulmonary Angiograms have images with intravenous contrast in the early arterial phase and it was considered a noncontrast CT Scan for the purposes of steatosis analysis. CT criteria for steatosis with intravenous contrast in the venous phase included liver attenuation 20 HU less than spleen attenuation [10]. CT Scans with intravenous contrast in the arterial phase or with heterogenous spleen attenuation were excluded. Liver attenuation was a mean of four measurements in areas 5 and 6. Spleen attenuation was a mean of a posterior and an anterior measurement.

### 2.5. Measurement of Abdominal Adipose Tissue

Waist circumference and subcutaneous fat depth were measured on the abdominal CT Scans. Waist circumference was measured at the level of the umbilicus. In scans where portion of the abdomen was outside of the image field, the contour of the waist circumference was estimated with a continuous arc. The subcutaneous fat depth was measured at three points: right and left midclavicular lines at the level of the umbilicus, as well as suprapubically at the level of iliac crest. The mean of these three values was calculated.

**Figure 2:**
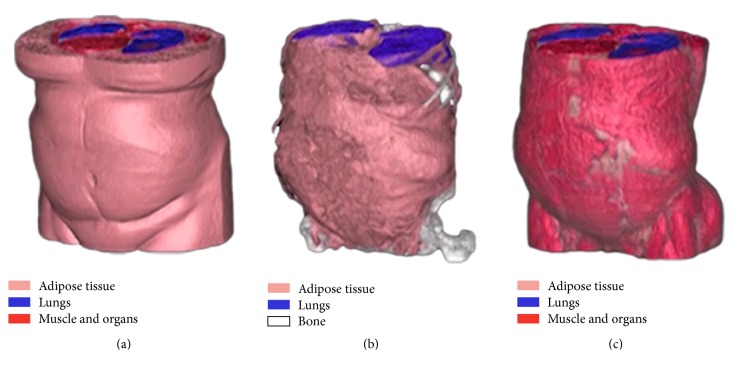
Reconstructed CT images demonstrating SAT (a) and VAT (b) volumes, as well as abdominal musculature (c). Muscle (red), lungs (blue), and bone (white) are also seen.

Semiautomated segmentation was performed on all patients using an adaptation of a previously validated method for fat distribution imaging [29]. CT images were loaded into the software by a trained observer and a subset of slices were selected for segmentation and volume measurement. For each patient, the superior boundary volume was selected as the axial slice where the dome of the liver was first visible and the inferior boundary where the top of the femoral heads was visible. Full volume adipose tissue was identified as any tissue within −205 to −25 HU range. Partial volume adipose, which was classified as tissue between −25 and 0 HU range and located on the boundary of full volume adipose, was also included as adipose. After tissue identification, adipose tissue was segmented into subcutaneous and visceral volumes, as shown in Figure 2. Nonvisceral adipose structures such as paravertebral and intermuscular were removed by a trained observer using tools available with the software. Finally, all pixels in the segmented adipose volumes were summed and converted to spatial volumes (cm^3^).

Two-tailed *t*-tests were performed using Excel for Mac 2011, version 14.4.1 (Microsoft). Statistics were performed using IBM SPSS statistics 2.0.

## 3. Results

### 3.1. Incidental Finding of Fatty Liver Disease

Over a 6-month period from January to July 2011, 1259 CT Scans of the thorax, abdomen, and pelvis were performed at the London Health Science Centre's (LHSC) Emergency Department (ED), of which 450 (35.7%) met the inclusion criteria. The 450 patients were comprised of 223 female and 227 male patients, with a mean age of 58.0 ± 18.2 (range 18–95) years. Of those 450 patients, the reading radiologists documented 48 patients (10.7%) as having the incidental finding of fatty liver disease. The 48 patients included 19 female and 29 male patients, with a mean age of 54.1 ± 13.9 (range 24–78) years.

Electronic chart reviews were performed on the 48 patients who had the incidental finding of fatty liver disease, to determine follow-up and assess initial blood work drawn during their emergency room visit (Table 1). Sixty-seven percent (*N* = 12/18) had elevated levels of alanine aminotransferase (ALT; normal < 33 units/L; range 9–64 units/L; mean 39 ± 17 units/L). Aspartate aminotransferase (AST; normal < 32 units/L) was elevated in 32% of patients (*N* = 8/25; mean 30 ± 12 units/L; range 10–63). Alkaline phosphatase (ALP; normal 35–104 units/L) was elevated in 4 of 27 patients (15%; mean 76 ± 26; range 38–129). Two of 6 patients (33%) had elevated gamma glutamyl transferase (GGT; normal < 61 units/L; mean 68 ± 58 units/L; range 12–153). Two of 48 patients (4%) had abnormal platelet counts (normal 150–400; mean 233 ± 62; range 104–360). Partial thromboplastin time (PTT; normal 25–39 seconds) and International Normalized Ratio (INR; normal 0.9–1.1) were increased in 0 of 32 (0%; mean 29 ± 2; range 23–36) and 4 of 32 (13%; mean 1.1 ± 0.1; range 0.8–1.3) patients, respectively.

### 3.2. Follow-Up of Patients with Fatty Liver Disease

One-year follow-up was assessed via electronic chart review and contact with the patient's family physician. None of the 48 patients with incidental findings of fatty liver had any subsequent documented follow-up bloodwork, imaging, testing, or clinic visits in the one year following their initial CT finding of fatty liver disease.

The family physicians of the 48 patients with incidental findings of fatty liver were contacted via telephone to determine if they were aware of the result of the LHSC ER CT Scan. We were unable to contact two of the 48 (4%) family physicians despite repeated attempts. Of the remaining 46 patients, 6 patients (13%) had no family physician and 34 patients (74%) had family physicians who were unaware. Six of the 46 patients (13%) had family physicians who were aware of the CT results; however none of the family physicians had performed further investigations or management of the finding.

### 3.3. Prevalence of Steatosis

Forty-five (2 with reported fatty liver disease and 43 without reported fatty liver disease) of the 450 patients were excluded from the HU analysis as the CT Scans had intravenous contrast visualized in the arterial phase. Of the remaining CT Scans, 168 had no intravenous (IV) contrast used, 44 had IV contrast visualized in the early arterial phase, and 193 had IV contrast visualized in the venous phase. One hundred of the 405 included patients (24.7%) met CT criteria for steatosis. Of the 46 patients with fatty liver disease reported by the radiologist who were included in the HU analysis, 40 patients (87.0%) fulfilled criteria for steatosis and 6 patients (13.0%) did not. Sixty of 359 patients (16.7%) without reported fatty liver disease met CT criteria for steatosis. A radiologist report of the incidental finding of fatty liver disease had a sensitivity of 40%, specificity of 98%, positive predictive value of 0.87, negative predictive value of 0.83, positive likelihood ratio of 20.3, and negative likelihood ratio of 0.6 when compared to CT criteria for steatosis.

### 3.4. Association of Steatosis with Waist Circumference and Abdominal Adipose Tissue

Waist circumference, as measured on CT, was significantly larger in patients with incidental finding of steatosis (*N* = 46) as compared with a cohort of patients with no reported steatosis (*N* = 97) (113.6 ± 11.4 cm versus 99.1 ± 13.9 cm, *P* < 0.001). Right periumbilical (38.8 ± 4.4 mm versus 24.5 ± 10.7 mm, *P* < 0.001), left periumbilical (38.8 ± 4.2 mm versus 24.1 ± 10.7 mm, *P* < 0.001), suprapubic (38.0 ± 6.2 mm versus 25.0 ± 11.6 mm, *P* < 0.001), and mean (34.3 ± 12.5 mm versus 24.4 ± 11.0 mm, *P* < 0.001) subcutaneous fat depth were significantly larger in patients with steatosis (*N* = 46) versus a cohort of those without steatosis (*N* = 98).

Two hundred and ninety-four patients had complete abdominal CT Scans available for measurement of adipose tissue volumes. Compared with patients who did not meet CT criteria for steatosis (*N* = 215), patients meeting CT criteria for steatosis (*N* = 79) had significantly greater abdominal subcutaneous adipose tissue (SAT) volume (13,726 ± 7,691 versus 9,863 ± 5,686 cm^3^, *P* < 0.0001), abdominal visceral adipose tissue (VAT) volume (9546 ± 5773 versus 6,644 ± 4,464 cm^3^, *P* < 0.0001), and total abdominal tissue (TAT) volume (23,349 ± 11,878 versus 18,071 ± 21,921 cm^3^, *P* = 0.04).

Linear regression comparing abdominal adipose tissue volumes with liver attenuation (Hounsfield Units) demonstrated a significant association with VAT (accounting for 26% of variation in liver attenuation; *P* < 0.001), SAT (accounting for 62% of variation, *P* < 0.001), and TAT (accounting for 47% of variation, *P* < 0.001).

## 4. Discussion

This study is the first to report Canadian prevalence of hepatic steatosis. With 24.7% of patients meeting CT criteria for steatosis, our study is above the global average of 20% [30] but below reported prevalence of 33.6% in the United States of America [31]. Canada does have a lower obesity rate than the United States of America [32] and thus rates of steatosis in Canada would be expected to be lower. This study provides an interesting Canadian perspective on the prevalence of steatosis.

Our study also demonstrates poor follow-up of an incidental finding of steatosis. Other studies have shown lack of follow-up of concerning incidental findings discovered on ER CT Scans in patients seen for renal colic and trauma [33, 34]. The lack of follow-up of steatosis in this study represents a missed opportunity to intervene earlier with these patients, as recommendations are clear on the evaluation of patients with incidentally discovered hepatic steatosis [35]. Guidelines recommend that patients with hepatic steatosis incidentally detected on imaging should be evaluated as though they have suspected NAFLD and worked up accordingly if they have symptoms or signs attributable to liver disease or have abnormal liver biochemistries (Strength 1, Evidence A) [35]. Twelve of the 18 (67%) patients with incidentally discovered steatosis who had liver enzymes drawn had an abnormal ALT, with their family doctor being aware of the incidentally found steatosis in only 3 (2 with normal ALT and 1 with abnormal ALT) of the 12 patients. Guidelines also recommend that, in patients with unsuspected hepatic steatosis in whom steatosis is incidentally detected on imaging and who lack any liver-related symptoms or signs and have normal liver biochemistries, it is reasonable to assess metabolic risk factors (e.g., obesity, glucose intolerance, and dyslipidemia) and alternate causes for hepatic steatosis such as significant alcohol consumption or medications (Strength 1, Evidence A) [35]. Regarding patients with normal liver enzymes and incidentally discovered steatosis, very few family physicians were aware of the incidental finding of steatosis (2 of 7 with normal ALT, 4 of 16 with normal AST, 3 of 23 with normal ALP, and 1 of 4 with a normal GGT) and no family physician was aware of guidelines for specific follow-up of these patients.

We show a significant relationship with waist circumference, visceral and subcutaneous fat measurements, and liver steatosis. One of the main limitations of diagnosing steatosis on CT is the inability to determine if the steatosis is reactive to infectious or inflammatory conditions, such as hepatitis or alcohol induced or secondary to metabolic syndrome. Although this correlation between fatty liver and increase in visceral and subcutaneous fat does not exclude confounding etiology, it certainly adds support to the hypothesis that visceral fat is central to the pathophysiology of NAFLD.

Radiologists significantly underreported the presence of steatosis on CT. Radiologists only reported 40% of the patients who met criteria for steatosis (for every patient correctly reported as having steatosis, there were 1.5 patients with steatosis that were unreported). Failing to report steatosis on CT is a missed opportunity to intervene in the lives of these patients. There were also six of the 46 patients reported to have steatosis who did not meet criteria for steatosis. Misdiagnosis of potential liver disease may have consequences, such as unnecessary parenchymal biopsies.

There are limitations to this study that must be taken into account when interpreting the results. There may be selection bias in the inclusion of the patients as patients who present to the ER during “on-call” hours may not be representative of the general population. As in any retrospective observational study, there may have been unmeasured confounders. We have no information on medications, diet, stress levels, physical activity, medical comorbidities, or other medical history or physical exam history. CT has difficulty differentiating between steatosis and steatohepatitis, as well as nonalcoholic fatty liver disease and nonalcoholic steatohepatitis. No histology is available to compare with CT findings. Although NAFLD is the most common cause of steatosis, there are other causes that we cannot exclude, including alcoholic liver disease (defined as >21 drinks per week for men and >14 drinks per week for women over at least a two-year period), hepatitis C (particularly genotype 3), and other less common causes of steatosis [35]. Pregnancy-related liver disease is ruled out, as patients are required to declare if they are pregnant and to have a negative *β*-HCG prior to CT. However, steatosis seen on imaging (no matter the cause) requires investigation and follow-up [35].

In summary, our findings suggest that 10.7% of patients receiving CT Scans in the ER have incidental findings of steatosis reported by the radiologist and that follow-up of these reports is lacking. Twenty-five percent of patients met CT criteria for steatosis, providing an estimate of the Canadian prevalence. Lack of follow-up of incidental findings of steatosis represents a missed opportunity for early detection and intervention to prevent further complications. More research is required to further characterize and understand the potential long term impact of recognition and follow-up of hepatic steatosis identified in this setting on the progression and outcome of the disease. Improving the knowledge among family physicians of this condition, its associated risk factors, and its management is important in identifying patients who require further investigations and management.

## Figures and Tables

**Table 1:** Blood work performed during the Emergency Department visit of the 48 patients with the incidental finding of fatty liver.

	AVG ± SD	Range	Abnormal values
ALT (normal < 33) *N* = 18	39 ± 17	9–64	67% *N* = 12

AST (normal < 32) *N* = 25	30 ± 12	10–63	32% *N* = 8

ALP (normal 35–104) *N* = 27	76 ± 26	38–129	15% *N* = 4

GGT (normal < 61) *N* = 6	68 ± 58	12–153	33% *N* = 2

INR (normal 0.9–1.1) *N* = 32	1.1 ± 0.1	0.8–1.3	13% *N* = 4

PTT (normal 25–39) *N* = 32	29 ± 2	23–36	0% *N* = 0

Platelet (normal 150–400) *N* = 48	233 ± 62	104–360	4% *N* = 2

## References

[1] Seidell J. C. (2005). Epidemiology of obesity. *Seminars in Vascular Medicine*.

[2] The World Health Organization (2000). Preventing and managing the global epidemic. *WHO Technical Report Series*.

[3] Sanyal A. J. (2002). AGA technical review on nonalcoholic fatty liver disease. *Gastroenterology*.

[4] Lall C. G., Aisen A. M., Bansal N., Sandrasegaran K. (2008). Nonalcoholic fatty liver disease. *American Journal of Roentgenology*.

[5] White D. L., Kanwal F., El-Serag H. B. (2012). Association between nonalcoholic fatty liver disease and risk for hepatocellular cancer, based on systematic review. *Clinical Gastroenterology and Hepatology*.

[6] Agopian V. G., Kaldas F. M., Hong J. C. (2012). Liver transplantation for nonalcoholic steatohepatitis: the new epidemic. *Annals of Surgery*.

[7] Dendl L. M., Schreyer A. G. (2012). Steatohepatitis—a challenge?. *Der Radiologe*.

[8] Lawrence D. A., Oliva I. B., Israel G. M. (2012). Detection of hepatic steatosis on contrast-enhanced CT images: diagnostic accuracy of identification of areas of presumed focal fatty sparing. *American Journal of Roentgenology*.

[9] Saadeh S., Younossi Z. M., Remer E. M. (2002). The utility of radiological imaging in nonalcoholic fatty liver disease. *Gastroenterology*.

[10] Jacobs J. E., Birnbaum B. A., Shapiro M. A. (1998). Diagnostic criteria for fatty infiltration of the liver on contrast-enhanced helical CT. *American Journal of Roentgenology*.

[11] Mahabadi A. A., Massaro J. M., Rosito G. A. (2009). Association of pericardial fat, intrathoracic fat, and visceral abdominal fat with cardiovascular disease burden: the Framingham Heart Study. *European Heart Journal*.

[12] Rosito G. A., Massaro J. M., Hoffmann U. (2008). Pericardial fat, visceral abdominal fat, cardiovascular disease risk factors, and vascular calcification in a community-based sample the framingham heart study. *Circulation*.

[13] Wells M., Croome K. M., Janik T., Hernandez-Alejandro R. M., Chandok N. M. (2014). Comparing outcomes of donation after cardiac death versus donation after brain death in liver transplant recipients with hepatitis C: a systematic review and meta-analysis. *Canadian Journal of Gastroenterology and Hepatology*.

[14] Boyko E. J., Fujimoto W. Y., Leonetti D. L., Newell-Morris L. (2000). Visceral adiposity and risk of type 2 diabetes: a prospective study among Japanese Americans. *Diabetes Care*.

[15] Goodpaster B. H., Krishnaswami S., Resnick H. (2003). Association between regional adipose tissue distribution and both type 2 diabetes and impaired glucose tolerance in elderly men and women. *Diabetes Care*.

[16] Kanaya A. M., Harris T., Goodpaster B. H., Tylavsky F., Cummings S. R. (2004). Adipocytokines attenuate the association between visceral adiposity and diabetes in older adults. *Diabetes Care*.

[17] Tulloch-Reid M. K., Hanson R. L., Sebring N. G. (2004). Both subcutaneous and visceral adipose tissue correlate highly with insulin resistance in African Americans. *Obesity Research*.

[18] Wagenknecht L. E., Langefeld C. D., Scherzinger A. L. (2003). Insulin sensitivity, insulin secretion, and abdominal fat: the Insulin Resistance Atherosclerosis Study (IRAS) Family Study. *Diabetes*.

[19] Ding J., Visser M., Kritchevsky S. B. (2004). The association of regional fat depots with hypertension in older persons of white and African American ethnicity. *American Journal of Hypertension*.

[20] Hayashi T., Boyko E. J., Leonetti D. L. (2004). Visceral adiposity is an independent predictor of incident hypertension in Japanese Americans. *Annals of Internal Medicine*.

[21] Sironi A. M., Gastaldelli A., Mari A. (2004). Visceral fat in hypertension: Influence on insulin resistance and *β*-cell function. *Hypertension*.

[22] Kobayashi H., Nakamura T., Miyaoka K. (2001). Visceral fat accumulation contributes to insulin resistance, small-sized low-density lipoprotein, and progression of coronary artery disease in middle-aged non-obese Japanese men. *Japanese Circulation Journal*.

[23] Lemieux S., Prud'homme D., Moorjani S. (1995). Do elevated levels of abdominal visceral adipose tissue contribute to age-related differences in plasma lipoprotein concentrations in men?. *Atherosclerosis*.

[24] Nicklas B. J., Penninx B. W. J. H., Ryan A. S., Berman D. M., Lynch N. A., Dennis K. E. (2003). Visceral adipose tissue cutoffs associated with metabolic risk factors for coronary heart disease in women. *Diabetes Care*.

[25] Fox C. S., Massaro J. M., Hoffmann U. (2007). Abdominal visceral and subcutaneous adipose tissue compartments: association with metabolic risk factors in the framingham heart study. *Circulation*.

[26] Targher G., Bertolini L., Poli F. (2005). Nonalcoholic fatty liver disease and risk of future cardiovascular events among type 2 diabetic patients. *Diabetes*.

[27] Yki-Järvinen H. (2005). Fat in the liver and insulin resistance. *Annals of Medicine*.

[28] Tilg H., Hotamisligil G. S. (2006). Nonalcoholic fatty liver disease: cytokine-adipokine interplay and regulation of insulin resistance. *Gastroenterology*.

[29] Jolly U. S., Soliman A., McKenzie C. (2013). Intra-thoracic fat volume is associated with myocardial infarction in patients with metabolic syndrome. *Journal of Cardiovascular Magnetic Resonance*.

[30] Vernon G., Baranova A., Younossi Z. M. (2011). Systematic review: the epidemiology and natural history of non-alcoholic fatty liver disease and non-alcoholic steatohepatitis in adults. *Alimentary Pharmacology and Therapeutics*.

[31] Szczepaniak L. S., Nurenberg P., Leonard D. (2005). Magnetic resonance spectroscopy to measure hepatic triglyceride content: prevalence of hepatic steatosis in the general population. *American Journal of Physiology—Endocrinology and Metabolism*.

[32] Vasan R. S., Pencina M. J., Cobain M., Freiberg M. S., D'Agostino R. B. (2005). Estimated risks for developing obesity in the Framingham Heart Study. *Annals of Internal Medicine*.

[33] Messersmith W. A., Brown D. F. M., Barry M. J. (2001). The prevalence and implications of incidental findings on ED abdominal CT scans. *American Journal of Emergency Medicine*.

[34] Munk M.-D., Peitzman A. B., Hostler D. P., Wolfson A. B. (2010). Frequency and follow-up of incidental findings on trauma computed tomography scans: experience at a level one trauma center. *The Journal of Emergency Medicine*.

[35] Chalasani N., Younossi Z., Lavine J. E. (2012). The diagnosis and management of non-alcoholic fatty liver disease: practice guideline by the American Gastroenterological Association, American Association for the Study of Liver Diseases, and American College of Gastroenterology. *Gastroenterology*.

